# The Role of Point of Care Ultrasound (PoCUS) in Orthopaedic Emergency Diagnostics

**DOI:** 10.7759/cureus.13046

**Published:** 2021-01-31

**Authors:** Jennifer Oluku, Attila Stagl, Kamalpreet S Cheema, Karmen El-Raheb, Richard Beese

**Affiliations:** 1 Trauma and Orthopaedic Surgery, Queen Elizabeth Hospital, London, GBR; 2 Clinical Radiology, Queen Elizabeth Hospital, London, GBR

**Keywords:** orthopedics and trauma, achilles tendon injury, occult fracture, joint effusion, pocus (point of care ultrasound)

## Abstract

Ultrasound has been described as the “stethoscope” of the radiologist; its ability to aid in clinical diagnosis with both static and dynamic imaging has allowed fast and accurate diagnosis. However, traditionally unlike a stethoscope, a large and bulky ultrasound machine made it difficult to use portably in a hospital environment where patients can be scattered across a hospital. With the development of innovative ultrasound technology, Point of Care Ultrasound (PoCUS) can readily be carried by a clinician to make a quick and timely diagnosis. In this review article we look at the uses of PoCUS within orthopaedic emergencies. Diagnosis in orthopaedics often requires further imaging beyond history taking, clinical examination and plain radiographs. In these cases PoCUS can be useful for ruling out occult fractures, diagnosing joint effusions and tendon ruptures. By aiding a speedy diagnosis, we can reduce unnecessary immobilisation, reduce inpatient stays, introduce early mobilisation and reduce harm to patients. With PoCUS becoming increasingly cheaper and more portable we feel this really can become the stethoscope of an orthopaedic surgeon.

## Introduction and background

Point of care ultrasound (PoCUS) is a powerful diagnostic tool which can improve the speed of reaching a diagnosis; it can remove uncertainty from a list of differential diagnosis and aid initiating treatment. Its use becomes more apparent in the emergency department where there are many scenarios with acute conditions which require time critical investigation and management. PoCUS has its use in less austere settings, where more complex diagnostics imaging is not readily available. Often referred to as the stethoscope of the radiologist, the use of PoCUS in combination with clinical history and examination has seen to improve accuracy and speed of diagnostic testing, minimising delays between presentation and the consequent initiation of definitive treatment.

Whilst there are barriers to the use of PoCUS, it does not appear to be due to a learning curve. A large retrospective study was performed assessing emergency medicine residents which looked at the learning curves of using PoCUS; it set out to identify performance plateaus for both image acquisition and interpretation of the residents. These findings were then compared to those of expert sonographers [1]. Residents were measured in nine areas which represented the core ultrasound applications as defined by American College of Emergency Physicians (ACEP). The study found that in all these areas there were progressive and gradual improvements usually followed by a plateau. They found that some scans were more difficult to master than others, however there were some scans that plateaus occurred at relatively low experience levels (27 scans). Residents were able to acquire skills to interpret soft tissue and endovaginal uterus the easiest, with sensitivity in the mid-90s. They concluded that educational performance benchmarks occur at varied points for image interpretation and image quality for different examination scan types and this information should be utilised when developing standards [1].

**Figure 1:**
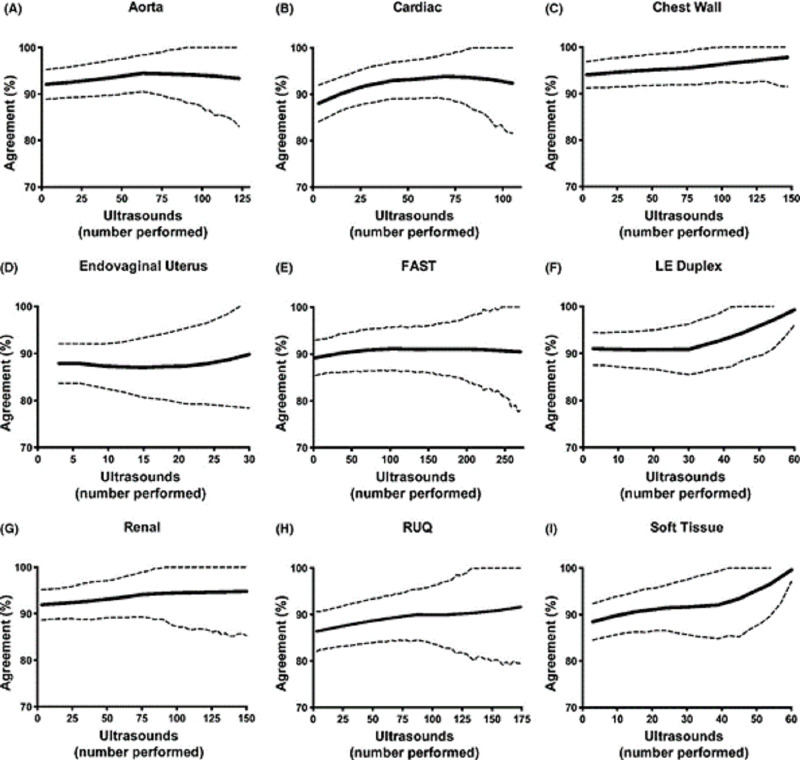
Image demonstrating learning curves as well as plateau points of various ultrasound scans surrounded by 95% confidence interval. The solid line represents the percentage agreement with an expert and the dotted line represents the 95% confidence interval. Figure from Blehar et al. 2015 [1] Original images authorised by respective owners.

The learning curve as well as the number of scans required to meet proficiency to a set standard is outlined in Figure 1.

This reflects in another study conducted by Chenkin et al.; 66 physicians completed a study in performing endotracheal intubation and identification of incorrectly placed oesophageal intubations. After a brief tutorial and two practice attempts emergency physicians were able to increase their score from a baseline test of 42.9% to 90.9% with a total error rate of 0.9%, with participants achieving an overall sensitivity of 98.3 and a specificity of 100%. The learning curve and skill acquisition using PoCUS is scan dependent and can require minimal training to become proficient [2].

## Review

PoCUS in practice

Ultrasound is not traditionally taught in the United Kingdom (UK) as part of the undergraduate training curriculum at medical school; however, this is not the case for other parts of the world. This may play a factor in its limited uptake within the UK, in addition to the lack of available trainers and training curriculum [3]. There has, however, been some adoption of its use in some specialties. One of the biggest adopters of PoCUS is acute and emergency physicians. In the trauma setting PoCUS has been used as a diagnostics tool for detecting intraperitoneal haemorrhage, pericardial tamponade pneumothorax as well as haemothorax - the extended Focused Assessment with Sonography for Trauma (FAST scan). A meta-analysis found that a positive fast scan for detecting intra-abdominal injury had a sensitivity of 74% and a specificity of 96%. In comparison to other techniques to identify intra-abdominal injury [4], its use has not just been limited to trauma but is extended to other acute presentations in the emergency department [5].

In the acute obstetric and gynaecology presentations, PoCUS is used in ruling out ectopic pregnancies and consequent need for surgical intervention. A meta-analysis compared the use of PoCUS to formal radiological or gynaecological ultrasound in ruling out ectopic pregnancy. There was strong evidence to support its use with significant heterogeneity within the pooled data [6]. A prospective observational study looked at the presence of fluid in the hepatorenal space (Morrison's pouch) - its identification signalling the likelihood of requiring operative management; nine out of 10 identified patients using PoCUS went on to require operative management after formally correlated investigations [7]. Chest physicians utilise PoCUS in performing thoracocentesis. Strongly recommended by the British Thoracic Society (BTS), studies show the accuracy of safe site insertion is increased by 26% with ultrasound [8].

PoCUS in occult fractures

Diagnosing bony fractures can be a diagnostic dilemma when treating patients in the emergency department. Fractures especially those which are undisplaced can be difficult to identify on plain radiographs alone. Soft tissue injuries can mimic fracture presentation and it can be difficult to rule out a fracture from clinical examination. Access to further imaging modalities such as computerized tomography (CT) as well as magnetic resonance imaging (MRI), is precious and often unavailable out of hours. Consequently, waiting for further imaging modalities can lead to unnecessary admissions, unnecessary immobilisation as well as a delay in treatment. In the case of paediatric patients further imaging may carry risks associated with radiation as well as non-compliance [8].

Traditionally, ultrasound was thought to be of no use in the examination of bone due to its density and it being highly reflective on sonography; however the highly vascular nature of bone means that when it is fractured, subperiosteal fluid can be seen with ultrasound and cortical disruptions or irregularities for diaphyseal fractures. In the case of intra-articular fractures, a haemarthrosis may be seen which can raise the suspicion of a fracture; conversely no fluid or cortical breaches is very specific in ruling out fractures. In the literature ultrasound has been shown to have a specificity of 94% and a negative predictive value of 97% [8].

**Figure 2:**
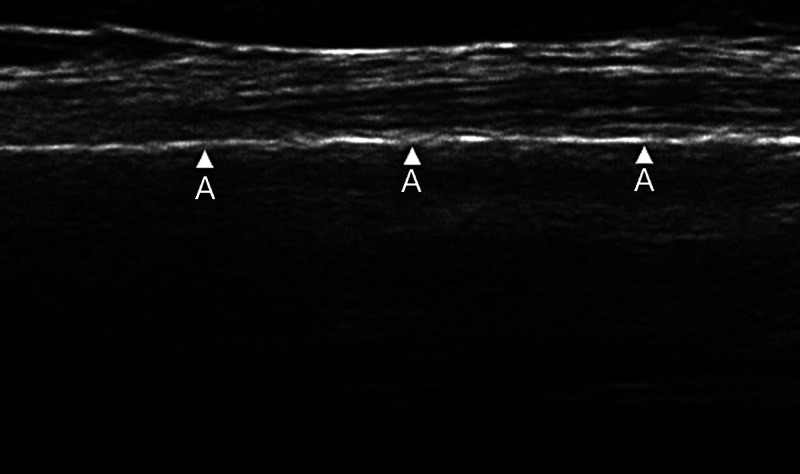
Ultrasound of intact tibial diaphyseal cortex as demonstrated by the arrowheads Ultrasound image from Qadi et al. 2020 [9] A- Intact continuous bony cortex Original images authorised by respective owners

An ultrasound demonstrating an intact cortex of bone is shown below in Figure 2. The bone is highly reflective to ultrasound waves and thus the bone cortex appears as a bright white line.

**Figure 3:**
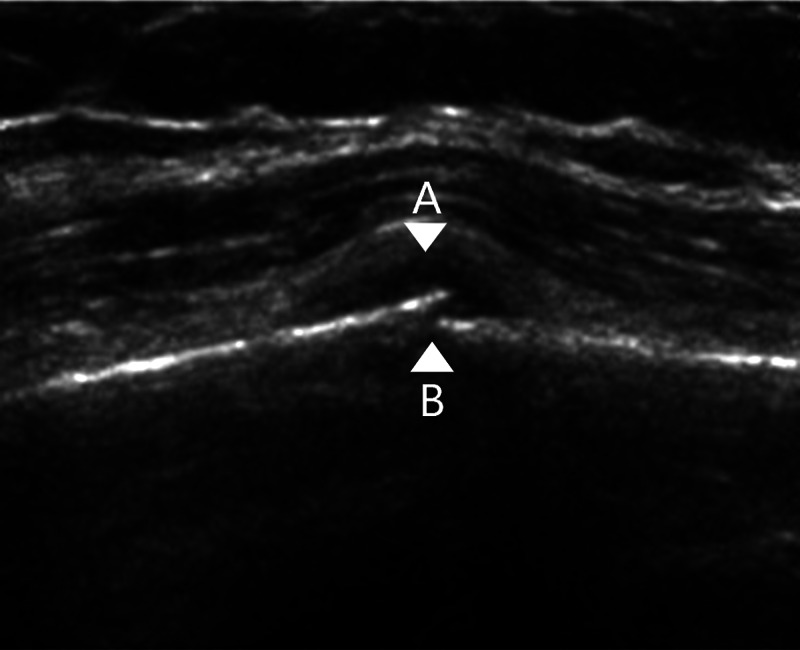
Ultrasound of a diaphyseal tibial fracture demonstrating a break in the cortex, periosteal haemtoma formation and periosteal lift. Ultrasound images from Qadi et al., 2020 [9] A- Periosteal haematoma B- Loss of continuity of the bony cortex and periosteal lift Original images authorised by respective owners

Signs of a fracture on an ultrasound scan can be seen in Figure 3 with a loss of continuity of the cortex, as well as a periosteal haematoma and periosteal lift.

PoCUS in tendon injury

Tendon injuries are an ideal use of PoCUS as most tendons are relatively superficial anatomical structures. Diagnosis can often be made by history and clinical examination alone, however, up to 20% of these ruptures can be missed in the initial hyperacute phase [10]. A delay in diagnosis will lead to a delay in treatment which in turn can lead to a difficult repair; this leads to a poorer function in performance and strength for the patient [10,11].

To assess for an Achilles tendon rupture, a linear transducer is used - as it has better anatomical resolution. In a normal tendon, the tendon fibres are oriented in a continuous fibrillar linear and regular pattern. In case of a ruptured tendon discontinuity of the fibres is seen, fibres may appear wavy, thickened or have an abrupt end, haematoma can also be visualised between the two ends. Secondary signs include hypoechogenic areas deep or within the tendon, oedema of the tissue or degeneration of the tendon; this can also be observed in sprains and tendinopathy. A systematic review found that there was 79.6%-100% sensitivity for ultrasound in detection of Achilles tendon rupture [12,13]. The spread in specificity was large within the review, however, two studies achieved perfect data with 100% specificity [12,13]. Ultrasound was also able to determine between full and partial thickness ruptures with 100% sensitivity and 83% specificity [12,13].

**Figure 4:**
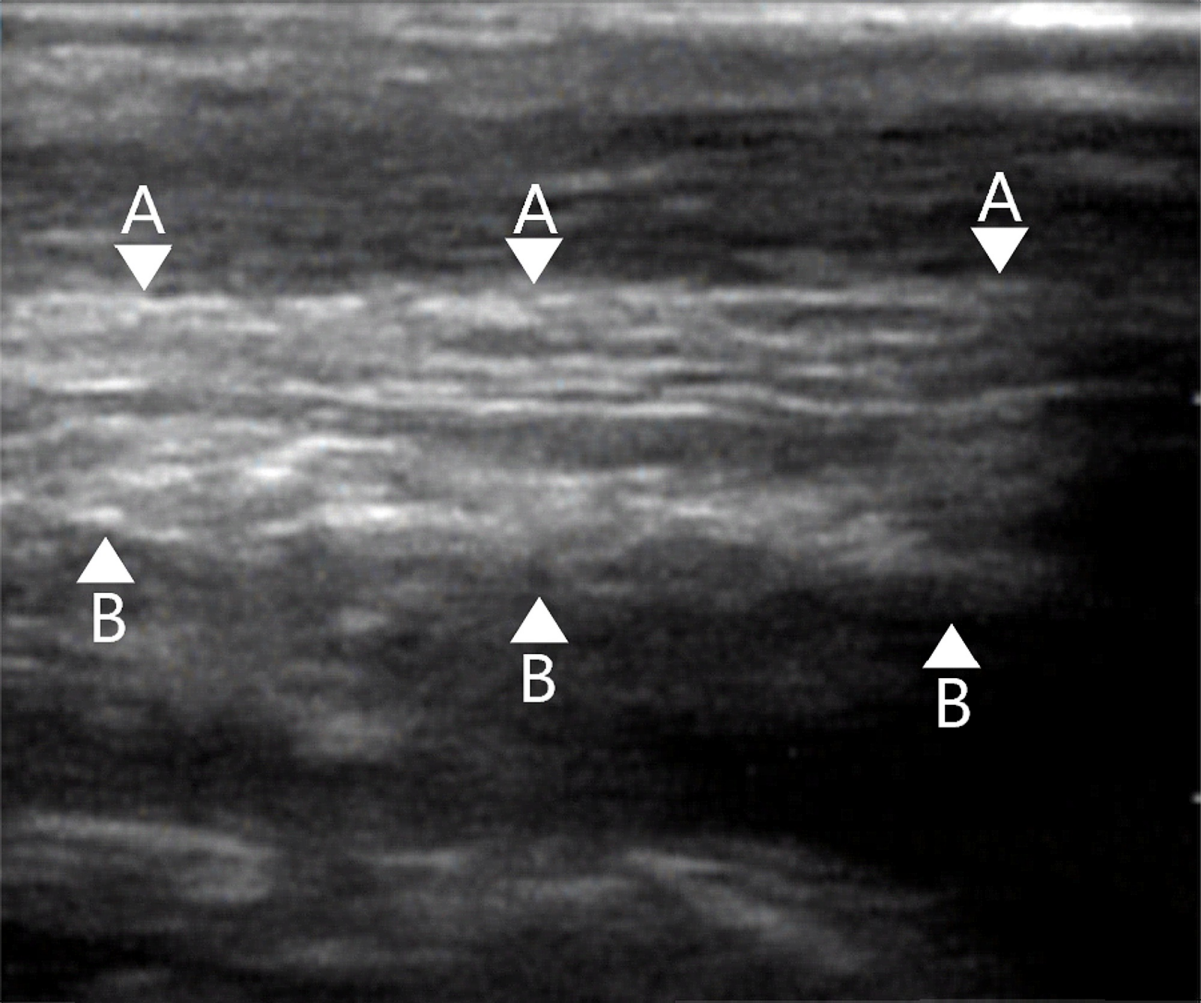
Ultrasound of a normal Achilles tendon in the longitudinal axis showing a continuous linear pattern which is fibrillar and hyperoechoic. Ultrasound images from Kopinksi and Davis, 2020 [14] A- Intact anterior aspect of Achilles tendon B- Intact posterior aspect of Achilles tendon Original images authorised by respective owners

**Figure 5:**
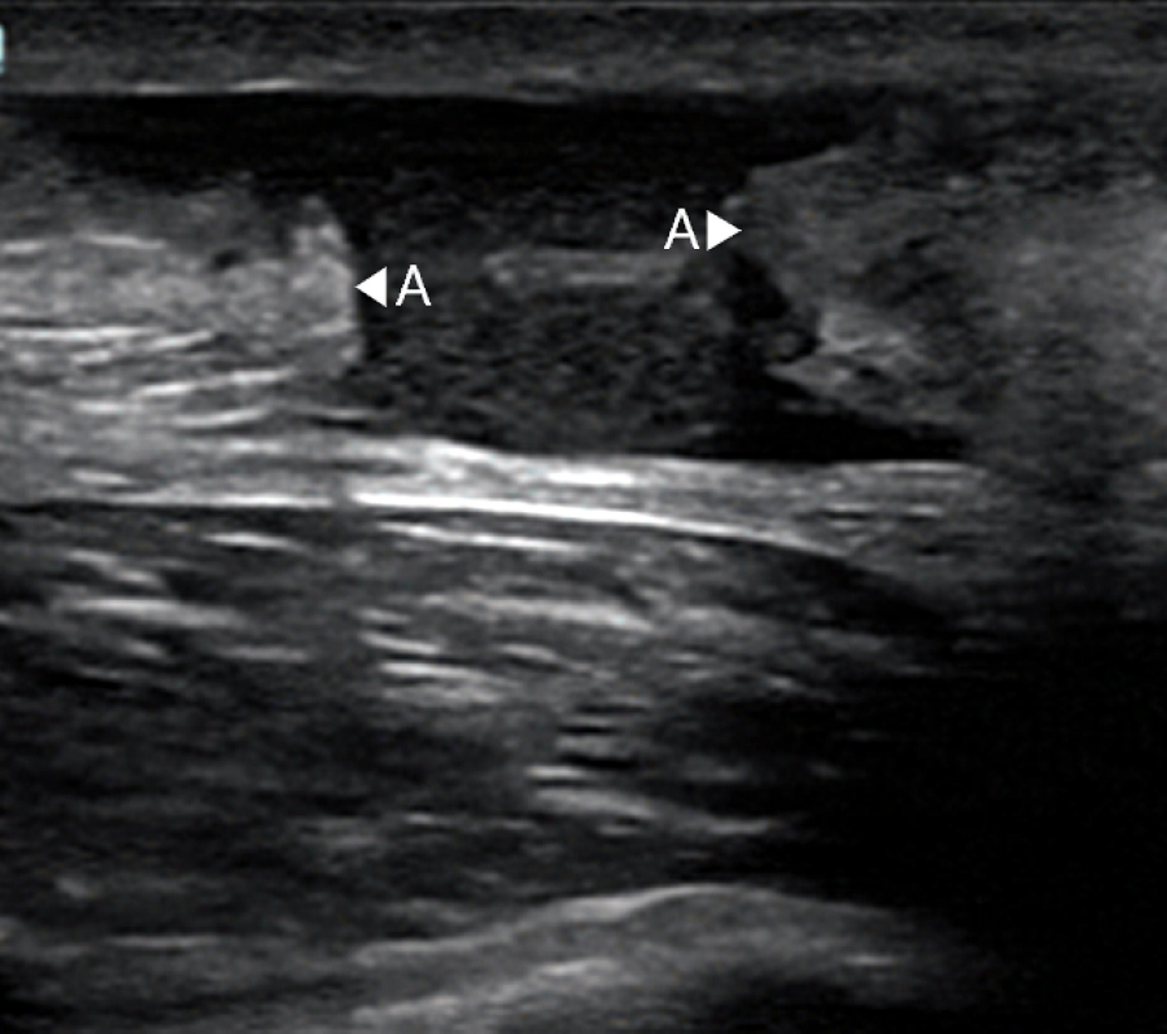
Demonstration of a full thickness Achilles tendon rupture in the longitudinal axis. Ultrasound image from Butterfield, 2020 [15] A- Disruption of Achilles tendon, visualisation of abrupt and thickened ends Original images authorised by respective owners

A normal Achilles tendon ultrasound scan in the longitudinal axis is shown in Figure 4. Whilst a full thickness Achilles tendon rupture can be seen in Figure 5.

PoCUS in joint effusions

Swollen and tender joints are a very common presentation in the emergency department. Differentiating between a septic arthritis, bursitis, a flare of a crystal or non-crystal arthropathy or a traumatic effusion can be difficult to identify just on clinical history and presentation alone.

PoCUS can be particularly helpful in adults presenting with atraumatic acute hip pain or a child presenting with hip pain, a limp or reluctance to bear weight. In the hip, it is particularly important to assess for septic arthritis. Septic arthritis is a medical emergency, if left untreated has a mortality of 10% [16], it can also cause joint destruction in a matter of hours. Therefore, diagnosis, investigation and intervention need to be carried in a timely fashion to decrease morbidity and mortality. Ultrasound and MRI is commonly used to determine the presence of a hip joint effusion; this is particularly key in the paediatric population where it can be difficult to obtain an appropriate history and examination.

Assessment of septic arthritis includes history, clinical examination, blood tests looking at inflammatory markers, as well as plain radiograph imaging. Plain radiographs are able to distinguish between traumatic injury or identify bony deformities; occasionally they can show widening of the joint space in large effusions. Ultrasound is considered the gold standard in diagnosis of hip effusions, it can determine the presence of an effusion, however it cannot distinguish if this effusion is infective or not.

To identify if an effusion is present, a high frequency linear probe (curvilinear probe if more depth is required) is used, the patient is placed in a supine position, the greater trochanter is identified and the femoral head located - an anterior synovial fluid collection greater than 5 mm, or more than a 2-mm difference in the contralateral hip is positive for an effusion [16].

**Figure 6:**
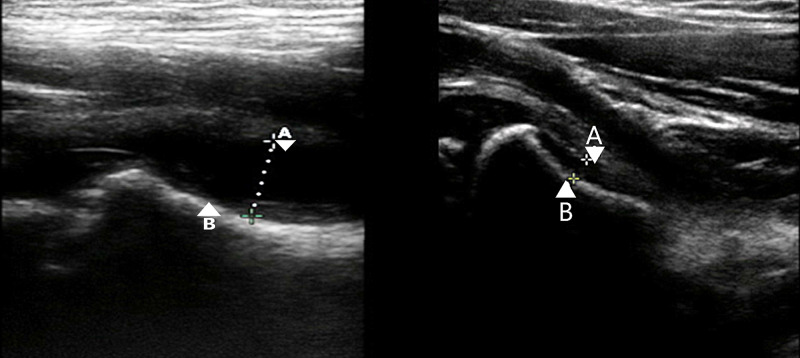
Ultrasound demonstrating a hip joint effusion in a paediatric patient is shown on the left. For comparison normal physiological joint fluid volume is shown on the right. Ultrasound images from Pade et al., 2020 [16] A – Capsule of hip joint B – Bony cortex of femur Dotted line - Depth of hip effusion Original images authorised by respective owners

An ultrasound scan showing a hip effusion is shown on the left in Figure 6. For comparison a normal volume of physiological joint fluid is shown on the right in Figure 6.

The current largest retrospective study in 2020 showed that the use of PoCUS expedites diagnosis of hip effusion and consequent management, in comparison to waiting for technician performed ultrasound within the radiology department. A statistically significant difference was found in time from presentation to ultrasound results and consequent arthrocentesis [(median time from first emergency physician contact to ultrasound of 68 (range 38.8-132) minutes, as compared to 208.5 (range 163.8-301.3) minutes for patients who received formal radiological department ultrasound (P = <0.001)] [17]. It was also highlighted that in the paediatric population, emergency physicians were also able to provide the sedation needed to perform these scans safely and effectively [17]. Vieira and Levy have shown that with limited focus training PoCUS can identify hip effusions in the paediatric population with a sensitivity of 85% and a specificity 100% [18]. Boniface et al. in their case series also found that PoCUS was able to identify hip effusion in their adult population for which their initial findings were correlated with the use of CT or MRI imaging [19].

Its effectiveness in detecting hip effusions in both the adult and paediatric population makes PoCUS an underutilised diagnostic tool (particularly in the adult population), which could potentially improve morbidity and mortality in these patients. In patients with hip pain post traumatic injury, PoCUS is able with 100% specificity and 65% sensitivity to identify abnormal sonographic findings - identifying patients who are suspicious of occult hip fracture [20]. Being able to identify those who are suspicious for having a fracture can then be triaged to have further imaging in the form of CT or MRI scan.

Benefits of efficient diagnosis

The use of PoCUS for patients presenting to the emergency department with a possible Achilles tendon rupture is beneficial in a number of ways. With high sensitivity and specificity PoCUS can determine if a tendon rupture is present or not. Normally based on clinical examination and history, all these patients would be managed with immobilisation and venous thromboembolism (VTE) prophylaxis until adequate imaging had been procured, i.e., formal ultrasound within the radiology department or MRI imaging - this could potentially pose significant delay in initiating appropriate management. In identifying these patients who do not have ruptures, this would prevent unnecessary immobilisation and the risks posed with VTE prophylaxis treatments; in addition to removing these risks, the cost ensued from more formal diagnostic tests is removed and the patient in turn can be managed more timely with physiotherapy and analgesia, instead of awaiting management inappropriately in a speciality clinic [12].

The ability to triage cases appropriately does not only reduce the risk posed to patients from unnecessary interventions, such as immobilisation and venous thrombosis prophylaxis - with the risks associated with these management, it also reduces the need for admission. The impact of delayed patient discharge can have massive consequences for the patient. Immobilisation in a hospital bed is associated with increased morbidity and mortality with some studies quoting up to 8.6% mortality [21,22]. Increased inpatient stays can lead to a deterioration in patient mental health and thus result in poor outcomes on activities of daily living on discharge [21]; there is also a cost to the hospital with the estimated cost of an extra day ranging from £200-£565 per patient per day - translating to a cost of more than £100,000 per year for a London-based ward of 30 beds. Members of the Multidisciplinary Team (MDT) are also impacted, with increased feelings of stress and disillusion as a direct result of the delays in the discharge process [21]. With urgent diagnosis with PoCUS we can reduce all these unnecessary risks and additional costs.

The use of PoCUS has been found to improve the doctor-patient relationship. Evidence shows that patient satisfaction is significantly increased. This is possibly as a consequence of the faster time to diagnosis and associated decreased length of stay; patients were also found to have better perceptions of the emergency physician as well as the physicians' skills and abilities in providing their medical care [23,24].

## Conclusions

PoCUS has really seen a rapid increase in its utilisation within the emergency department in the last decade; however, its utilisation by orthopaedics at the point of care, is still very limited. PoCUS has demonstrated great ability to help with diagnostics in orthopaedics, especially for joint effusions, tendon ruptures as well as occult fractures. This efficiency in diagnosis can initiate prompt definitive treatment as well as prevent unnecessary immobilisation. The use of PoCUS can help triage the need for further imaging in cases of traumatic joint effusion, or sonographic abnormalities such as periosteal lift or cortical disruption for extra-articular fractures. In combination with clinical history and examination, this will reduce inpatient stays as well as reduce the number of more formal and expensive CT and MRI imaging. With PoCUS becoming more portable as well as cheaper, it is with confidence we state that PoCUS can really become the stethoscope of the orthopaedic surgeon.
